# CryptoDex: A randomised, double-blind, placebo-controlled phase III trial of adjunctive dexamethasone in HIV-infected adults with cryptococcal meningitis: study protocol for a randomised control trial

**DOI:** 10.1186/1745-6215-15-441

**Published:** 2014-11-12

**Authors:** Jeremy Day, Darma Imran, Ahmed Rizal Ganiem, Natriana Tjahjani, Retno Wahyuningsih, Robiatul Adawiyah, David Dance, Mayfong Mayxay, Paul Newton, Rattanaphone Phetsouvanh, Sayaphet Rattanavong, Adrienne K Chan, Robert Heyderman, Joep J van Oosterhout, Wirongrong Chierakul, Nick Day, Anatoli Kamali, Freddie Kibengo, Eugene Ruzagira, Alastair Gray, David G Lalloo, Justin Beardsley, Tran Quang Binh, Tran Thi Hong Chau, Nguyen Van Vinh Chau, Ngo Thi Kim Cuc, Jeremy Farrar, Tran Tinh Hien, Nguyen Van Kinh, Laura Merson, Lan Phuong, Loc Truong Tho, Pham Thanh Thuy, Guy Thwaites, Heiman Wertheim, Marcel Wolbers

**Affiliations:** Wellcome Trust Major Overseas Programme Vietnam, Oxford University Clinical Research Unit, Ho Chi Minh City, Vietnam; Cipto Mangunkusum Hospital, Jakarta, Indonesia; Hasan Sadikin Hospital, Bandung, Indonesia; RSKO Drug Dependence Hospital, Jakarta, Indonesia; Laos Oxford Mahosot Wellcome Trust Research Unit, Mahosot Hospital, Vientiane, Laos; Malawi-Liverpool-Wellcome Trust, Clinical Research Programme, University of Malawi College of Medicine, Blantyre, Malawi; Mahidol Oxford Tropical Medicine Research Unit, Mahidol University, Bangkok, Thailand; MRC/UVRI Uganda Research Unit on AIDS, Entebbe/Masaka, Uganda; Nuffield Department of Population Health, University of Oxford Health Economics Research Centre, Oxford, UK; Wellcome Trust Tropical Centre Liverpool, Liverpool, UK; Hospital for Tropical Diseases, Ho Chi Minh City, Vietnam; National Hospital for Tropical Diseases, Hanoi, Vietnam; Bach Mai Hospital, Hanoi, Vietnam; Cho Ray Hospital, Ho Chi Minh City, Vietnam; National Hospital for Tropical Diseases Oxford University Clinical Research Unit, Hanoi, Vietnam; Department of Parasitology, Indonesia Christian University, School of Medicine, Jakarta, Indonesia; Department of Parasitology, Faculty of Medicine, University of Indonesia, Jakarta, Indonesia; Dignitas International, Zomba, Malawi

## Abstract

**Background:**

Cryptococcal meningitis (CM) is a severe AIDS-defining illness with 90-day case mortality as high as 70% in sub-Saharan Africa, despite treatment. It is the leading cause of death in HIV patients in Asia and Africa.

No major advance has been made in the treatment of CM since the 1970s. The mainstays of induction therapy are amphotericin B and flucytosine, but these are often poorly available where the disease burden is highest. Adjunctive treatments, such as dexamethasone, have had dramatic effects on mortality in other neurologic infections, but are untested in CM. Given the high death rates in patients receiving current optimal treatment, and the lack of new agents on the horizon, adjuvant treatments, which offer the potential to reduce mortality in CM, should be tested.

The principal research question posed by this study is as follows: does adding dexamethasone to standard antifungal therapy for CM reduce mortality? Dexamethasone is a cheap, readily available, and practicable intervention.

**Method:**

A double-blind placebo-controlled trial with parallel arms in which patients are randomised to receive either dexamethasone or placebo, in addition to local standard of care. The study recruits patients in both Asia and Africa to ensure the relevance of its results to the populations in which the disease burden is highest. The 10-week mortality risk in the control group is expected to be between 30% and 50%, depending on location, and the target hazard ratio of 0.7 corresponds to absolute risk reductions in mortality from 30% to 22%, or from 50% to 38%. Assuming an overall 10-week mortality of at least 30% in our study population, recruitment of 824 patients will be sufficient to observe the expected number of deaths. Allowing for some loss to follow-up, the total sample size for this study is 880 patients. To generate robust evidence across both continents, we aim to recruit roughly similar numbers of patients from each continent. The primary end point is 10-week mortality. Ethical approval has been obtained from Oxford University’s Tropical Research Ethics Committee (OxTREC), and as locally mandated at each site.

**Trial registration:**

International Standard Randomised Controlled Trial Number: ISRCTN59144167 26-July-2012

## Background and rationale

### Background

Cryptococcal meningitis (CM) is estimated to cause 625,000 deaths every year, most occurring within 3 months of diagnosis [1]. It is the leading cause of death in HIV patients in Asia and Africa, affecting 3.2% of the HIV-infected population per year [1]. The incidence in these regions is the highest in the world; in Africa, more deaths are estimated to be due to CM than to tuberculosis [1]. The 90-day case-fatality rate is up to 55% in Asia and 70% in Africa [1].

Despite improvements in access to HIV care, the WHO estimates that HIV/AIDS will be the leading cause of disease in middle- and low-income countries by 2015, and models suggest that, even if 80% access to HIV treatment is achieved by 2012, there will be 6.5 million AIDS deaths p.a. by 2030 [2]. Thus, CM is likely to remain a significant health burden for the foreseeable future.

No major advance has occurred in the treatment of cryptococcal meningitis since the 1970s. The mainstays of induction therapy are drugs that are more than 50 years old (amphotericin B and flucytosine), although these are often poorly available where the disease burden is highest. Although amphotericin therapy is undoubtedly superior to fluconazole monotherapy, amphotericin combination therapy has only recently been shown to reduce mortality when compared with amphotericin monotherapy. Whereas effective antifungal therapy is the key, adjunctive treatments, which have been seen to have dramatic effects on mortality in other neurologic infections, are untested in cryptococcal meningitis. Given the high death rates in patients receiving current optimal treatment, and the paucity of new agents on the horizon, adjuvant treatments offer the greatest potential to reduce mortality in CM.

This study aims to reduce the death rate from CM. The principal research question is as follows: does adding dexamethasone to standard antifungal therapy for CM reduce mortality? In this double-blind placebo-controlled trial (DBRCT) patients will be randomised to receive either dexamethasone or placebo. Dexamethasone is a cheap, readily available, and practicable intervention.

This multicentre study recruits patients in both Asia and Africa to ensure the relevance of the study results in the populations in which the disease burden is highest.

### Treatment of CM

Successful treatment of CM depends on effective antifungal therapy and successful management of complications, notably increased intracranial pressure (see Appendix 3). Antifungal treatment schedules for cryptococcal meningitis are not globally uniform but are affected by drug availability, costs, and human resources. The Infectious Diseases Society of America convenes an international panel to draw up treatment guidelines, most recently published in 2010, and the WHO currently has guidelines in development that will be specifically aimed at management in resource-poor countries. These conform closely to the IDSA guidelines. Treatment generally consists of a period of induction therapy by using high-dose or combination antifungal therapy (usually for 2 weeks), followed by a period of consolidation therapy of 8 weeks with fluconazole. After this time, provided that the patient has responded to treatment, secondary prophylaxis using lower-dose fluconazole is given to prevent disease relapse. It is generally considered safe to stop secondary prophylaxis if ARV therapy has resulted in suppression of the plasma viral load and immune reconstitution with recovery of the CD4 cell count to >100 cells/μl has lasted for at least 6 months).

Consistent with the local practices and the WHO and IDSA guidelines, in this study, patients will receive antifungal therapy consisting of amphotericin B (1 mg/kg/day) combined with fluconazole, 800 mg/day for 2 weeks, followed by fluconazole 800 mg/day for a further 8 weeks before switching to secondary prophylaxis [3, 4].

### Rationale for adjuvant treatment with dexamethasone

Several mechanisms exist through which dexamethasone may modify disease outcome in cryptococcal meningitis. Current IDSA guidelines suggest that corticosteroids may be beneficial in cryptococcal meningitis in patients who have cryptococcomas with mass effect, acute respiratory distress syndrome, or IRIS [4]. However, corticosteroids have never been tested in a randomised controlled trial. Of note, 150 of 381 patients in the ACTG trial of combination antifungal therapy in cryptococcal meningitis received steroids during the study. The Graybill (2000) *post hoc* analysis of these patients has some serious limitations (1997) [5, 6]. Steroids were prescribed at the discretion of the attending physician, and the reasons for prescription are unclear. The Graybill analysis was limited to just 41 of the 150 steroid recipients, and did not report their impact on mortality. Clinical success (defined as stabilisation or improvement at 2 weeks) was lower in the 41 steroid recipients analysed (66% versus 86%). Apparently worse mycologic outcomes in these 41 patients are impossible to interpret, because no adjustments were made for the antifungal therapy received, fungal load, or clinical severity at baseline, all of which are known to be important outcome predictors [7, 8]. The exclusion from the analysis of the vast majority of patients who received steroids means that it is impossible to draw robust conclusions about their effect and underlines the need for a trial.

In contrast with the article by Graybill, a study in HIV-uninfected patients with *C. gattii* meningitis found a 10-fold reduction in blindness in those who received steroids [9]. Moreover, in tuberculous meningitis (TBM), a disease that shares pathophysiologic features with CM, dexamethasone has been shown to improve outcome (RR of death, 0.69; 95% CI, 0.52 to 0.92; *P* =0.01) [10]. In this RCT, conducted in Vietnam, 545 patients were recruited, of whom 98 were HIV infected. In addition to the reduced risk of death, fewer adverse events were found in patients receiving dexamethasone [10].

### Potential mechanisms of action dexamethasone in CM

#### Antiinflammatory action

Cryptococcal meningitis is a chronic granulomatous meningitis, with foci of inflammation in the basilar meninges, cerebral mass lesions, and a lymphocytic cerebrospinal fluid (CSF). The basilar meningitis leads to impairment of resorption of CSF, and this, along with physical blockage of CSF drainage by yeast cells and conglomerations of capsule, is believed to contribute to the increased intracranial pressure seen in the disease [11]. The immune response in the mouse model mimics that seen in AIDS patients [12]. The effect of antiinflammatory agents has been tested in this model. Reduction of inflammation prolongs survival in mice infected with a lethal dose of *C. neoformans*[13]. Dexamethasone increases median survival from 19 to 26 days (*P* <0.05), a benefit preserved when dexamethasone is combined with amphotericin B [14]. Dexamethasone does not interfere with the pharmacologic properties of amphotericin or fluconazole [14, 15]. Moderation of the inflammatory response is postulated to be through microglial cells, a key in the immune response to CM [16].

#### Reduction of cerebral oedema and brain swelling

Cerebral oedema is a key feature of CM, and cryptococcal capsule has been shown directly to induce cerebral oedema [17]. Vascular endothelial growth factor (VEGF) is a potent inducer of vascular permeability and angiogenesis, which has been implicated in the pathogenesis of brain oedema [18, 19]. Elevated levels are seen in both the CSF and blood of HIV patients with cryptococcal meningitis, and cryptococcal capsule has been shown to induce VEGF production by monocytes, neutrophils, and peripheral blood mononuclear cells in a dose-dependent manner [18, 20]. *In vitro*, this induction is significantly downregulated by dexamethasone [18]. Downregulation occurs at a range of concentrations consistent with those achieved in human dosing. A rat model of brain oedema suggests that the effect of steroids on oedema is mediated through the inhibition of VEGF [21]. Corticosteroids reduce vascular permeability and limit oedema, and inhibition of VEGF may explain the beneficial effect of steroid therapy in TBM [10, 22].

#### Reduction of intracranial pressure

Increased intracranial pressure (ICP) is frequent in CM and is an important cause of mortality [5]. Dexamethasone reduces increased ICP in models of other brain infections [23–25]. The mechanisms are not clear, but through attenuation of inflammation, cerebral oedema, and restoration of the blood/brain barrier, it is plausible that dexamethasone may reduce increased ICP in CM.

#### Modification of cerebral vasculitis

Cerebral vasculitis, frequently described in infections, is a recognised feature of CM [26–29]. The pathophysiology of neurologic vasculitis is relatively well understood: the neurologic features arise principally through ischaemia and infarction secondary to inflammation [30]. In infections, the vascular insult may be mediated through any or all of vascular wall invasion, immune complexes, or cryoglobulins. Of these, both endothelial invasion and immune complex disease are described in cryptococcosis [31, 32].

Steroids are an important therapeutic option in primary cerebral vasculitides, and their beneficial effect in TBM may be through an antivasculitic action [30]. Attenuation of the vasculitis seen in CM may improve outcome.

### Potential harms of dexamethasone

Reported side effects of dexamethasone are well described and similar to those of other corticosteroids. Side effects include dysglycaemia, changes in mood, Cushing-like syndrome, gastrointestinal bleeding, immunosuppression, hypertension, and secondary hypoadrenalism. Side effects are more likely with higher doses (dexamethasone ≥16 mg/kg/day) and longer courses of treatment [33]. Dexamethasone is frequently prescribed for patients with intracranial pathology, including infectious diseases, and in addition, data on adverse events are available from large RCTs of dexamethasone that have been completed in tuberculous meningitis (TBM) and acute bacterial meningitis (BM) [10, 34–36]. In the BM trials, the duration of steroid therapy was 4 days, and in TBM, 6 to 8 weeks. In all these trials, adverse events, including potentially life-threatening adverse events such as gastrointestinal bleeding, were rare and were no more common in patients receiving dexamethasone compared with placebo.

In this study, patients will receive dexamethasone 0.3 mg/kg/day, reducing weekly over 6 weeks. This is the lower dose that was used in the TBM trial for patients with Grade I disease [10]. The risk and severity of any adverse events must be considered in the context of the high mortality seen in cryptococcal disease. Notably, dexamethasone has no mineralocorticoid effect and is not associated with hypokalaemia [37].

#### Immunosuppression

Corticosteroids are immunosuppressive. It is possible that they may slow yeast clearance from CSF, and increase the risk of other infections. However, animal studies suggest that dexamethasone improves yeast clearance from CSF [14]. Even if the rate of yeast clearance is reduced, this may not be detrimental. In this study, we will measure the rate of clearance of yeast from CSF (see Appendix 1). Patients with CM are profoundly immunosuppressed: the median CD4 count in Vietnamese patients is 16 cells/μl (which compares with a median CD4 count of 42 in Vietnamese HIV patients with tuberculous meningitis) [38]. Given this, whether dexamethasone will significantly increase the risk of other opportunistic infections (OIs) is not clear. The incidence of other OIs is a secondary end point of the study. Patients in the study will receive chemoprophylaxis against *Pneumocystis jirovecii* and other opportunistic infections, in line with national guidelines. Patients will be screened for TB at study entry as part of normal care.

#### Secondary hypoadrenalism

Adrenal suppression is a recognised risk in patients receiving corticosteroid therapy for prolonged periods or in higher doses. With a treatment course of the length in this study, adrenal suppression will be short lived and is prevented through the gradual reduction in dose, as stipulated in the protocol. Patients must be provided with an information card detailing the importance of taking the steroids as per the study protocol, and so that other doctors who attend them will be aware of their prescription. Steroid information cards must be provided in the local language. Steroids will be administered in a reducing dose according to best medical practice.

#### Hyperglycaemia

Reversible hyperglycaemia is a recognised side effect of corticosteroids. Hyperglycaemia is uncommon in patients receiving dexamethasone in this dose, with elevated fasting glucose occurring in fewer than 1% of patients and equally frequently amongst TBM patients receiving active drug or placebo [10]. A review of the adverse effects of corticosteroids in severe sepsis found an increased absolute risk of hyperglycaemia of 5.6% [39].

#### Cushing-like syndrome

A Cushing- type syndrome can develop in people receiving prolonged steroid therapy, with weight gain, redistribution of body fat, and acne. However, in the dose and duration given in this study, development of Cushing syndrome is unlikely. Moreover, it is reversible on treatment cessation.

#### Gastrointestinal bleeding

Data from TBM, bacterial meningitis, and sepsis suggest that corticosteroids do not increase the risk of gastrointestinal bleeding when used in these dosages and durations [10, 35, 36, 39].

#### Hypertension, salt retention, and hypokalaemia

These effects of corticosteroids are due to their mineralocorticoid action. Because dexamethasone belongs to the glucocorticoid group, the risk of these side effects is negligible.

### Overview of the trial

A multicentre double-blind randomised placebo-controlled trial of adjunctive treatment with dexamethasone in adults with HIV-associated cryptococcal meningitis

**Study aim:** To reduce mortality from cryptococcal meningitis in HIV-infected adult patients

**Intervention:** Dexamethasone in a reducing dose over the first 6 weeks of treatment

**Randomisation:** 1:1 study-intervention versus placebo, by using variably sized blocks and stratified by site

**Primary end point:** Survival during the first 10 weeks after randomisation

**Antifungal therapy:** Amphotericin B, 1 mg/kg/day + fluconazole 800 mg/day for 2 weeks, followed by fluconazole, 800 mg/day for 8 weeks, followed by secondary prophylaxis with fluconazole, 200 mg/day

**Secondary end points:** Survival during 6 months after randomisation, disability, rates of fungal clearance, rates of visual impairment, rates of IRIS, rates of new AIDS-defining illnesses, frequency of grade 3, grade 4, or serious adverse events, rates of increased intracranial pressure, time to new neurologic events, health economics analysis.

**Sample size:** 880 patients (roughly equal numbers from Asia and Africa)

**Participating countries:** Uganda, Malawi, Vietnam, Thailand, Indonesia, Laos

**Study sponsor:** Oxford University

**Study funding:** The UK Department for International Development, The Wellcome Trust (UK), and the Medical Research Council (UK) Joint Global Health Trials Scheme

**Figure 1:**
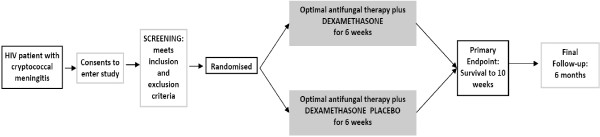
**Trial flow diagram.**

**Figure 2:**
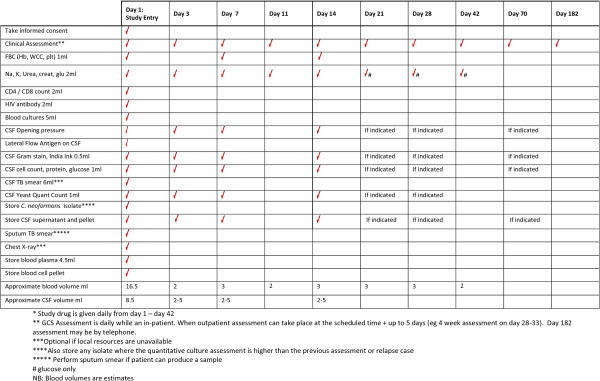
**Trial flow chart.**

**Study duration (recruitment and follow-up):** 3 years (Figures 1 and 2).

## Study aims

### Primary aim

To investigate the effect of dexamethasone adjunctive therapy on 10-week survival in HIV-infected adult patients with cryptococcal meningitis

### Secondary aims

The secondary aims are to determine the effect of adjuvant treatment with dexamethasone on survival at 6 months, disability at 10 weeks and 6 months, the rate of sterilisation of cerebrospinal fluid (CSF), the frequency of grades 3, 4, and serious adverse events, the incidence of IRIS, the incidence of other opportunistic infections, repeated treatment for cryptococcal meningitis, the presence of visual deficit at 10 weeks, and health economics. In addition, the effect of steroids on survival by continent will be assessed.

## End points

### Primary end point

The primary outcome is overall survival until 10 weeks after randomisation.

### Secondary end points

#### Survival until 6 months after randomisation

Relapse occurs in cryptococcal meningitis. Most cases occur within 6 months of diagnosis. We will collect survival data at 6 months (alive versus date of death) to ensure that any survival benefit at 10 weeks is sustained at 6 months, and not negated by, for example, a higher relapse rate in patients receiving steroids.

#### Disability at 10 weeks and 6 months

Neurologic disability will be assessed by using the modified Rankin score and the Two Simple Questions and classified as good, intermediate, severe disability, or death, as previously described (Table 1) [10].

**Table 1:** **Outcome and disability grading: “two simple questions” and rankin score**

The two simple questions		
Does the patient require help from anybody for everyday activities? *(For example eating, drinking, washing, brushing teeth, going to the toilet.)*	Yes/no	Yes = Poor outcome
Has the illness left you with any other problems?	Yes/no	Yes = Intermediate outcome
		No = Good outcome
**The modified rankin scale**		
**Grade**	**Description**	
0	No symptoms	
1	Minor symptoms not interfering with lifestyle	
2	Symptoms that lead to some restriction in lifestyle, but do not interfere with the patients’ ability to look after themselves	
3	Symptoms that restrict lifestyle and prevent totally independent living	
4	Symptoms that clearly prevent independent living, although the patient does not need constant care and attention	
5	Totally dependent, requiring constant help day and night	

Grade 0: Good outcome, Grade 1 or 2: Intermediate outcome, Grade 3-5: poor outcome.

Disability is an expected consequence of cryptococcal meningitis, including blindness, deafness, and other focal neurological deficits. In addition to reduced death rate, patients receiving dexamethasone may also have lesser (or higher) rates of these disabilities. The Rankin score and the Two Simple Questions are well-validated measures of the degree of disability in stroke survivors, and have been used frequently to measure disability after neurologic infections.

#### Rate of CSF sterilisation during the first 2 weeks

It is conceivable that dexamethasone could slow the rate of CSF sterilisation, although this is not seen in the animal model. In a subset of patients, we will measure the repeated fungal burdens in CSF over the first 2 weeks of treatment, model the rate of decline, and determine the effect of dexamethasone on fungal clearance. We will also determine whether the rate of CSF sterilisation is predictive of mortality in both study arms, based on joint modelling of longitudinal fungal counts and mortality. Specifically, the longitudinal model will be a mixed model with fixed effects for the intercept, time (that is, the slope), and a treatment-time interaction and random (bivariate normal) effects for the intercept and the slope. The Cox survival model will have shared random effects with the longitudinal model.

#### Adverse events

Comparison of the proportion of patients with any grade 3 or 4 adverse event and of serious adverse events between treatment groups will form an important part of the study analysis, to determine the safety of the intervention.

#### Rate of IRIS until 10 weeks

We will model the rate of IRIS over time with a cause-specific hazards model, taking into account the competing risk of prior death. CM-related IRIS will be defined as per the recent proposed definition (see Appendix 2) [40].

#### Time to new AIDS-defining illnesses or death until 10 weeks

AIDS-defining illnesses will be defined as per the WHO classification (see Table 2).

**Table 2:** **WHO clinical staging for HIV/AIDS**

**Clinical stage 1**	Asymptomatic
	Persistent generalised lymphadenopathy (PGL)
	Performance scale 1: asymptomatic, normal activity
**Clinical stage 2**	Weight loss, <10% of body weight
	Minor mucocutaneous manifestations (seborrheic dermatitis, prurigo, fungal nail infections, recurrent oral ulcerations, angular cheilitis)
	Herpes zoster, within the last 5 years
	Recurrent upper respiratory tract infections (for example, bacterial sinusitis)
	And/or performance Scale 2: symptomatic, normal activity.
**Clinical stage 3**	Weight loss, >10% of body weight
	Unexplained chronic diarrhoea, >1 month
	Unexplained prolonged fever (intermittent or constant), >1 month
	Oral candidiasis (thrush)
	Oral hairy leukoplakia
	Pulmonary tuberculosis, within the past year.
	Severe bacterial infections (for example, pneumonia, pyomyositis)
	And/or Performance scale 3: bedridden, <50% of the day during the last month
**Clinical stage 4**	HIV wasting syndrome, as defined by CDC^1^
	*Pneumocystis carinii* pneumonia
	Toxoplasmosis of the brain
	Cryptosporidiosis with diarrhoea, >1 month
	Cryptococcosis, extrapulmonary
	Cytomegalovirus (CMV) disease of an organ other than liver, spleen, or lymph nodes
	Herpes simplex virus (HSV) infection, mucocutaneous >1 month, or visceral of any duration
	Progressive multifocal leukoencephalopathy (PML)
	Any disseminated endemic mycosis (for example, histoplasmosis, coccidioidomycosis)
	Candidiasis of the oesophagus, trachea, bronchi, or lungs
	Atypical mycobacteriosis, disseminated
	Nontyphoid *Salmonella* septicaemia
	Extrapulmonary tuberculosis
	Lymphoma
	Kaposi sarcoma (KS)
	HIV encephalopathy, as defined by CDC^2^
	And/or Performance scale 4: bedridden, >50% of the day during the last month

(Note: Both definitive and presumptive diagnoses are acceptable).

^1^HIV wasting syndrome: weight loss of >10% of body weight, plus either unexplained chronic diarrhoea (>1 month), or chronic weakness and unexplained prolonged fever (>1 month).

^2^HIV encephalopathy: clinical finding of disabling cognitive and/or motor dysfunction interfering with activities of daily living, progressing over weeks to months, in the absence of a concurrent illness or condition other than HIV infection that could explain the findings.

#### Visual deficit at 10 weeks

Retrospective data from HIV-uninfected patients with meningitis due to *Cryptococcus gattii* suggest that steroids may have a profound effect in reducing visual loss; 10% of Vietnamese HIV patients have visual impairment at 10 weeks [41]. We will compare the incidence of blindness and other visual deficits between treatment groups. Visual deficit will be assessed by using a simple 6-point scale (see Table 3).

**Table 3:** **Visual assessment**

Visual assessment – record the best performance	
**Function**	**Score**
Normal	1
Blurred	2
Finger counting	3
Movement perception	4
Light perception	5
No light perception	6

#### Time to new neurologic event or death until 10 weeks

A neurologic event is defined as a decrease in Glasgow coma scale score by ≥2 points for ≥2 days from the highest previously recorded Glasgow coma scale score (including baseline) or the occurrence of any of the following adverse events: cerebellar symptoms, coma, hemiplegia, paraplegia, seizures, cerebral herniation, new-onset blindness or deafness, or cranial nerve palsy.

#### Longitudinal measurements of intracranial pressure during the first 2 weeks

Intracranial pressure (ICP) will be measured at study entry, days 3, 7, and 14, and if clinically indicated (depending on local practice). The main outcomes are longitudinal ICP measurements until day 14, and we will model the effect of dexamethasone on ICP based on a joint model for longitudinal and survival data similar to the model described for rate of CSF sterilization, above.

Clinical need and local practice will determine the frequency of lumbar punctures after day 14 and clinician’s diagnoses of increased ICP based on the presence of headache, nausea, diurnal and postural variation, relief with lumbar puncture, and presence of papilloedema will be somewhat subjective.

#### Antifungal treatment intensification or retreatment for cryptococcal meningitis in the 6 months after randomisation

Relapse occurs in patients with cryptococcal disease. All patients will be receiving either treatment doses of antifungal therapy or secondary prophylaxis doses during the 6-month period of follow-up. Although all patients will be encouraged to return to the study hospital in the event of illness during the 6-month follow-up period, it is possible that, because of their location, some patients will undergo treatment elsewhere where diagnostic facilities may be less developed. This episode may only be identified at the 6-month follow-up period. For this reason, a pragmatic definition of relapse will be used. This is defined as either intensification of antifungal therapy above that according to the study antifungal schedule, or readmission for treatment of cryptococcal disease. The main outcome measure will be the cause-specific hazard of relapse, taking into account the competing risk of death.

#### Health economics

If effective, the low cost of dexamethasone makes it promising as a cost-effective intervention in low-income settings. To assess this formally, a cost-effectiveness analysis will be conducted in collaboration with the Health Economics Research Centre, University of Oxford. The objective of the analysis will be to estimate the incremental cost-effectiveness ratio (ICER), expressed as cost per life year gained and cost per Quality Adjusted Life Year (QALY) gained and per Disability Adjusted Life Year (DALY) averted of dexamethasone treatment compared with standard antifungal therapy for cryptococcal meningitis (CM).

The analysis will collect information during the study on resources used and direct and indirect costs, including health care costs (treatments, medications, consultations, initial and subsequent hospitalisations), and patient/family incurred costs (out-of-pocket costs, employment). Health-care resources used will be obtained primarily from trial case record forms. Unit costs will be obtained in each country from participating centres and national sources. Information on patient-employment status and costs will be obtained from patients by a small number of questions administered at recruitment and again at the final follow-up (10-week) visit. The main measures of effectiveness will be (a) life years gained (b) quality-adjusted life years gained (QALYs), estimated by using the EQ-5D instrument in official English, Vietnamese, and Thai languages in its three-level version, and (c) disability-adjusted life years (DALYs) averted, which measures both years of life lost because of premature mortality and years lived with disability. Life years gained will be based on the primary outcome measure of survival at 10 weeks, extrapolated by using best estimates of longer-term survival in each country for HIV-infected adults. QALY and DALY estimates will adjust the life-year estimates by taking into account secondary end points, including blindness, deafness, and other neurologic disabilities that are important sequelae of cryptococcal disease. QALY adjustment will be done by using EQ-5D-3 L responses, and DALY adjustment by using clinical judgment to align recorded morbidity with DALY states. The use of both approaches will provide internal validity checks and maximise opportunities to present relevant information to decision makers.

As unit costs, absolute risks, life expectancy, and other variables are likely to differ substantially across regions, separate cost-effectiveness estimates will be produced for Asia and Africa; however, a trial-wide estimate of effectiveness will be applied unless clear evidence exists of heterogeneity in effect across regions. All estimates of costs, outcomes, and cost-effectiveness will be reported with full recognition of uncertainty, including cost-effectiveness acceptability curve and sensitivity analyses around key parameters, including unit costs, long-term life expectancy, and disability adjustment.

The economic evaluation is concerned primarily with estimation of cost and outcome differences and cost-effectiveness rather than hypothesis testing, and a power calculation for a ratio statistic is likely to be highly uncertain, especially as the DALY/QALY measure (the denominator) will itself be a composite of survival and quality/disability adjustment. The proposed size of the study (880 patients) should permit cost differences of 12% or greater to be reliably detected by assuming a coefficient of variance of 60%.

## Methods

### Study design

A randomised, double-blind, placebo-controlled trial with two parallel arms: dexamethasone versus placebo during the first 6 weeks of treatment. This multicentre study will recruit patients who offer informed consent across Asia and Africa, in Malawi, Uganda, Laos, Thailand, Indonesia, and Vietnam. The study is pragmatic, designed to maintain relevance through trialing adjuvant treatment with dexamethasone in the context of the best standard of locally available care.

### Dexamethasone-treatment dose

Dexamethasone will be given in a reducing dose according to body weight (Table 4):

**Table 4:** **Dexamethasone-reducing regimen**

Period	Dexamethasone Dose	Timing
Week 1	0.3 mg/kg iv	Once daily
Week 2	0.2 mg/kg iv	Once daily
Week 3	0.1 mg/kg po	Once daily
Week 4	3.0 mg total/day po	Once daily
Week 5	2.0 mg total/day po	Once daily
Week 6	1.0 mg total/day po	Once daily
Week 7 onwards	Stops	

This dose is identical to the dose used in patients with Grade 1 tuberculous meningitis and has been shown to have a low rate of side effects [10]. Dexamethasone/placebo will be administered intravenously, during the period of intravenous antifungal treatment, and orally once antifungal treatment is administered orally. Treatment will be weight-dosed to the nearest half milligram. With the exception of the first dose, which should be given with the first dose of antifungal therapy, dexamethasone should be given in the morning.

## Study population

All HIV-infected adult patients with a diagnosis of CM presenting to the study centres will be eligible to enter the study, subject to meeting the inclusion/exclusion criteria and giving informed consent.

### Trial location

The study will recruit patients at sites in Vietnam, Indonesia, Thailand, Laos, Uganda, and Malawi.

### Inclusion criteria

- Age ≥18 years
- HIV antibody positive
- Cryptococcal meningitis defined as a syndrome consistent with CM and one or more of
  - ○ positive CSF India ink (budding encapsulated yeasts),
  - ○ *C. neoformans* cultured from CSF or blood,
  - ○ positive cryptococcal antigen Lateral Flow Antigen Test (LFA) in CSF

Informed consent to participate given by patient or acceptable representative

### Exclusion criteria

- Pregnancy
- Active gastrointestinal bleeding (defined as vomiting blood or melena stool in the previous week)
- Currently receiving treatment for CM and having received ≥1 week of anti-CM therapy
- Known allergy to dexamethasone
- Current steroid use defined as
  - currently receiving the equivalent of prednisolone 40 mg/day or more
  - currently receiving steroid therapy (any dose) for more than 3 weeks (except topical steroids, which are permitted)
- Concurrent condition for which corticosteroids are indicated because of proven benefit (such as severe *Pneumocystis* pneumonia (pO_2_ < 70 mm Hg) or tuberculous meningitis)
- Renal failure (defined as creatinine >3 × ULN, despite adequate hydration)

## Study procedures

### Recruitment

The target population for study screening is any patient known or suspected to have CM. To enter the study, patients must be confirmed to have CM according to the definition in the inclusion criteria. Screening procedures will adapt to the standard of care of each setting to ensure that study-related procedures are performed only after informed consent has been obtained. According to the clinical care of the treating hospital, patients suspected of having CM will undergo

- an IMMY lateral-flow cryptococcal antigen test (LFA) on serum or plasma,
  AND/OR
- blood culture for *Cryptococcus*
  AND/OR
- a lumbar puncture with an IMMY lateral-flow cryptococcal antigen test, and/or microscopic examination of CSF, and/or culture of CSF.
- When the results of the IMMY lateral-flow test (plasma, serum, or CSF), or CSF microscopy, or blood or CSF culture are available, study staff may consider whether the patient should be approached regarding the study. Only patients 18 years or older who are not known to be pregnant and who have evidence of cryptococcal disease from one of the specified tests will be approached.

A member of study staff will invite the patient to discuss the details of the study. If the patient is judged by the staff to be unfit or unable to give informed consent, an acceptable representative will act on the patient’s behalf for the following procedures. The study staff will give the patient/representative a copy of the informed-consent form and explain the details of the study, including the study procedures, risks and benefits, financial and confidentiality considerations, treatment alternatives, and how to obtain more information. The study staff will invite the patient/representative to ask questions and will endeavour to ensure that she or he understands the information given. The study staff will then ask the patient/representative to consider study participation. Those who refuse consent will be treated as per the best available standard care and will not have any study-related procedures performed.

Those who consent to the study will sign and date two copies of the informed-consent form. The study staff will also sign and date the two copies.

If the patient/representative is illiterate, a witness who is not a member of the study staff will be present during the informed-consent discussion. The informed-consent form will be read to the patient/representative in the presence of the witness. If the patient/representative agrees to participate, the form will be signed and dated by the witness.

Consenting patients will be screened for eligibility.

### Screening

Only patients who are 18 years or older, who are not known to be pregnant, and who have at least one of positive CSF or blood/serum LFA test, positive blood or CSF culture, or positive CSF microscopy will be consented. Consenting patients will undergo the following screening procedures/tests. In the case of an unconscious patient, information will be obtained from a knowledgeable relative or caregiver.

- Medical history will be taken, including: (a) signs and symptoms consistent with cryptococcal meningitis, (b) allergy to dexamethasone, and (c) history of corticosteroid use and antifungal therapy.
- Patient will be checked for signs of active gastrointestinal bleeding.
- All women of child-bearing age will have a urine or blood pregnancy test.
- Creatinine level
- HIV status will be confirmed from clinical history or testing as per standard of care.
- A lumbar puncture will be performed on all patients to obtain CSF. CSF will be tested by: (a) India ink stain or equivalent, (b) culture, and (c) lateral-flow cryptococcal antigen test. Cultured isolates will be stored for subsequent studies.
- If a lumbar puncture was done recently (within 48 hours) for clinical care, and volume of fresh CSF remains available for these tests (stored according to established SOPs), the lumbar puncture need not be repeated.
- If the patient underwent a recent lumbar puncture, an additional puncture will be performed (provided no contraindications exist) if any of the following are true: (a) uncertainty exists regarding the microbiologic diagnosis, (b) increased intracranial pressure is suspected, (c) the previous puncture was >2 days before, and no effective treatment has been given, and (d) it is required for standard care. If none of these is true or if the patient refuses further lumbar puncture, the patients may be randomised without additional lumbar puncture, provided they are eligible for the study.
- If the lumbar puncture was not recent, it will be repeated (provided no contraindications exist) to confirm the diagnosis, and to determine CSF pressure and fungal burden.

All lumbar punctures require verbal or written consent according to local standard clinical practice. Failure to consent according to local practice is a contraindication to the procedure.

**Figure 3:**
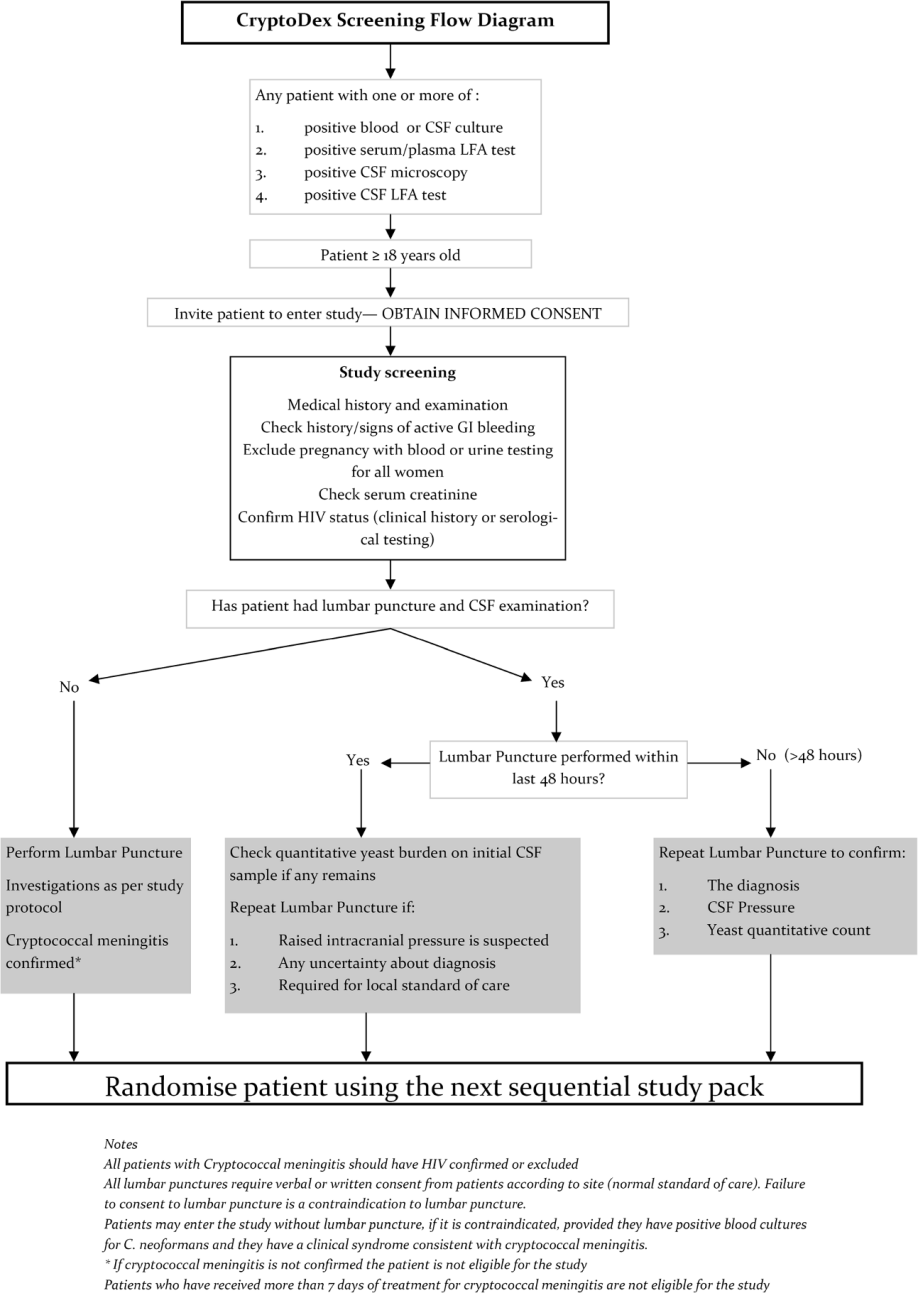
**Screening-study flow diagram.**

When all inclusion and exclusion criteria are verified, eligible patients will be randomised to treatment.Patients who are determined to be ineligible will be withdrawn from the study, and the reason recorded. Patients withdrawn from the study will be treated according to the best available standard care. Screening flow is illustrated in Figure 3.

The number of patients who do meet inclusion/exclusion criteria, but are not enrolled in the study will be recorded.

### Randomisation

Randomisation will be 1:1 to either dexamethasone or identical placebo. Patients will be stratified by hospital of enrolment. Enrolment logs specific to each site will be used to assign patients to the next available sequential number. The assigned number will correspond to a blinded, sealed, treatment pack containing dexamethasone for injection and dexamethasone tablets, or visually identical placebos. Treatment packs will be prepared centrally by an unblinded study pharmacist and distributed to the sites in batches, as required. Only central study pharmacists who hold the master randomisation list will know the contents of each pack. This list will be accessed only in the case of emergency unblinding, authorised by an investigator or designee, as per standard operating procedures. At each site we will use block randomisation with variable block lengths of size 4 (with probability 0.75) or 6 (with probability 0.25), respectively. Stratification by site will minimise the effect of any differences in patient or health care characteristics by ensuring that nearly equal numbers of patients receive either of the two treatment arms at each site. Drug appearance and administration schedules will be identical to maintain blinding amongst the attending physicians and nurses.

## Patient management

### Initial evaluation

On admission, all patients will have a full clinical assessment and examination.

Study-entry laboratory tests will be performed as per the study schedule in section Treatment of CM.

A baseline chest radiograph will be performed. A CT or MRI brain scan will be performed if evidence is found of increased intracranial pressure or focal neurologic abnormalities according to local practice and resources.

### Other treatment

#### Antifungal treatment

All patients will receive antifungal treatment consisting of 2 weeks of intravenous amphotericin B, 1 mg/kg/day, combined with high-dose fluconazole (800 mg/day), followed by fluconazole 800 mg/day for 8 weeks (10 weeks in total; see Appendix 4 and Appendix 5). This is locally feasible and consistent with recent guidelines [3, 4]. After the 10-week period of therapy, all patients, provided they have responded to treatment, will receive long-term secondary prophylaxis with fluconazole, 200 mg/day. Modification of antifungal therapy will be made according to the patient’s needs and the judgment of the attending physician. Any changes to antifungal therapy will be recorded in the case-record form. The cost of antifungal treatment (including secondary prophylaxis until 6 months after randomisation) will be covered by existing local financial support or trial finances.

#### Antiretroviral therapy

All patients must to be referred to local HIV services as soon as practicable, preferably while still admitted to hospital, to ensure that they have access to locally available HIV services, including counselling and ARVs.

It is not clear when antiretroviral therapy should be started in CM. Large studies are currently under way to answer this question. Early initiation may offer faster immune reconstitution benefiting survival, or may increase the risk of IRIS and drug toxicities, adversely affecting survival. In the absence of reliable data, most physicians would recommend starting ARVs after 4 weeks of antifungal treatment, provided the patient has made a good response. The date that ARVs are started (or stopped) in patients in the study will be recorded.

#### Opportunistic infection prophylaxis

Most patients will be profoundly immunosuppressed and should receive prophylactic therapy against other opportunistic infections, such as daily trimethoprim-sulfamethoxazole, in accordance with local guidelines and practices.

### Hospital admission

Intravenous amphotericin B is administered for 14 days, necessitating hospital admission during this period.

### Clinical monitoring

Patients will have daily GCS and review, as per standard care, until discharge from hospital. The decision to discharge patients from the hospital is at the attending physician’s discretion and is based on the clinical status of the patient. After discharge, patients will be seen weekly until 4 weeks, at 6 weeks, and at 10 weeks. If the exact visit day is not feasible, scheduled visits can occur at up to 5 days after the stipulated time to account for weekend and holidays (for example, the 4-week review should occur on day 28 to 33 after randomisation, and week 10 visit on day 70 through 75). Patients will be monitored closely for

- Death: the date of death and cause will be recorded
- New neurologic events (onset of new focal neurologic signs or decrease in Glasgow coma scale score of ≥2 points for ≥2 days, after >7 days clinical stability or improvement after randomisation)
- Drug-related adverse events
- New or recurrent AIDS-defining illnesses (see Table 5)

**Table 5:** **Presumptive and definitive criteria for AIDS-defining events**

	Presumptive criteria	Definitive criteria
**Constitutional disease**		
HIV wasting syndrome	Unexplained involuntary weight loss >10% from baseline PLUS persistent diarrhoea with ≥2 liquid stools/day for >1 month OR chronic weakness OR persistent fever >1 month. Should exclude other causes such as cancer, TB, MAC, cryptosporidiosis or other specific enteritis	
**Infections**		
Aspergillosis, other invasive	CXR abnormality compatible with aspergillosis PLUS invasive mycelia consistent with Aspergillus on lung biopsy or positive culture of lung tissue or positive culture of sputum	CXR abnormality compatible with aspergillosis PLUS invasive mycelia consistent with Aspergillus on lung biopsy PLUS positive culture of lung tissue or positive culture of sputum
Bartonellosis	Clinical evidence of bacillary angiomatosis or bacillary peliosis PLUS positive silver stain for bacilli from skin lesion or affected organ	Clinical evidence of bacillary angiomatosis or bacillary peliosis PLUS positive culture or PCR for *Bartonella quintana* or *Bartonella henselae*
Candidiasis of bronchi, trachea or lungs	None	Macroscopic appearance at bronchoscopy or histology or cytology (not culture)
Candidiasis, oesophageal	Recent onset retrosternal pain on swallowing PLUS clinical diagnosis or oral candidiasis by cytology (not culture) PLUS clinical response to treatment	Macroscopic appearance at endoscopy or histology or cytology (not culture)
Coccidiodomycosis, disseminated or extrapulmonary	None	Histology or cytology, culture or antigen detection from affected tissue
Cryptococcosis, meningitis or pulmonary	None	Histology or cytology/microscopy, culture or antigen detection from affected tissue
Cryptosporidiosis	None	Persistent diarrhoea >1 month, histology or microscopy
CMV retinitis	Typical appearance on fundoscopy of discrete patches of retinal whitening, associated with vasculitis, haemorrhage, and necrosis, confirmed by ophthalmologist	None
CMV end-organ disease	None	Compatible symptoms plus histology or detection of antigen from affected tissue
**Infections**	**Presumptive criteria**	**Definitive criteria**
CMV radiculomyelitis	Leg weakness and decreased reflexes or syndrome consistent with cord lesion presenting subacutely over days to weeks. CT/MRI shows no mass lesion. CSF shows >5 WBC with >50% polymorphs and positive CMV PCR, antigen or culture	None
CMV meningoencephalitis	Rapid (days to <4 weeks) syndrome with progressive delirium, cognitive impairment, ± seizures and fever (often with CMV disease elsewhere) CT/MRI may show periventricular abnormalities.	Rapid (days to <4 weeks) syndrome with progressive delirium, cognitive impairment, ± seizures and fever (often with CMV disease elsewhere) CT/MRI may show periventricular abnormalities and CSF PCR positive for CMV
HSV mucocutaneous ulceration	None	Persistent ulceration for >1 month, plus histology or culture or detection of antigen or HSV PCR positive from affected tissue
HSV visceral disease (for example, oesophagitis, pneumonitis	None	Symptoms, plus histology or culture or detection of antigen or HSV PCR positive from affected tissue
VZV multidermatomal	≥10 typical ulcerated lesions affecting at least two noncontiguous dermatomes plus response to an antiviral active against VZV unless resistance is demonstrated	≥10 typical ulcerated lesions affecting at least two noncontiguous dermatomes plus culture or detection of antigen or VZV PCR-positive from affected tissue
Histoplasmosis, disseminated or extrapulmonary	None	Symptoms plus histology or culture or detection of antigen from affected tissues
Isosporiasis	None	Persistent diarrhoea for >1 month, histology or microscopy
Leishmaniasis, visceral	None	Symptoms plus histology
Microsporidiosis	None	Persistent diarrhoea for >1 month, histology or microscopy
MAC, and other atypical mycobacteriosis	Symptoms of fever, fatigue, anaemia or diarrhoea plus acid-fast bacilli seen in stool, blood, body fluid, or tissue but not grown on culture and no concurrent diagnosis of TB except pulmonary	Symptoms of fever, fatigue, anaemia or diarrhoea plus culture from stool, blood, body fluid, or tissue
Tuberculosis, pulmonary	Symptoms of fever, dyspnoea, cough, weight loss, fatigue plus acid-fast bacilli seen in sputum, lavage, or lung tissue, not grown in culture, plus responds to standard TB treatment	Symptoms of fever, dyspnoea, cough, weight loss, fatigue plus positive TB culture or PCR from sputum, bronchial lavage, or lung tissue
Tuberculosis, extrapulmonary	Symptoms, plus acid-fast bacilli seen from affected tissue or blood but not grown in culture, concurrent diagnosis of pulmonary TB or responds to standard TB treatment	Symptoms, plus positive TB culture or PCR from affected tissue
Nocardiosis	Clinical evidence of invasive infection plus microscopic evidence of branching, Gram-positive, weakly acid-fast bacilli from affected tissue	Clinical evidence of invasive infection plus positive culture from blood or affected tissue
*Penicillium marneffei* disseminated	Characteristic skin lesions plus response to antifungal therapy for penicilliosis (in an endemic area)	Culture from a nonpulmonary site
*Pneumocystis* pneumonia (PCP)	Symptoms, any CXR appearance and CD4 count <200, negative bronchoscopy if treated for PCP for >7 days, no bacterial pathogens in sputum, and responds to PCP treatment	Microscopy or histology
Extrapulmonary pneumocystis	None	Symptoms plus microscopy or histology
Recurrent bacterial pneumonia	Second pneumonic episode within 1 year, new CXR appearance, symptoms and signs, diagnosed by a doctor	Second pneumonic episode within 1 year, new CXR appearance, detection of a pathogen
Progressive multifocal leukoencephalopathy (PML)	Symptoms and brain scan consistent with PML and no response to treatment for toxoplasmosis	Symptoms and brain scan consistent with PML and positive JC virus PCR in CSF or histology
*Rhodococcus equi* disease	None	Clinical evidence of invasive infection plus culture of organism from blood or affected tissue
Recurrent *Salmonella* septicaemia	None	Second distinct episode, culture confirmed
Cerebral toxoplasmosis	Symptoms of focal intracranial abnormality or decreased consciousness, and brain scan consistent with lesion(s) having mass effect or enhancing with contrast, and either positive toxoplasma serology or response to treatment clinically and by scan	Histology or microscopy
Extra-cerebral toxoplasmosis	None	Symptoms plus histology or microscopy
**Neoplasms**		
Kaposi sarcoma (KS)	Typical appearance without resolution. Diagnosis should be made by an experienced HIV clinician	Histology
Cervical carcinoma, invasive	None	Histology
Lymphoma, primary cerebral	Symptoms consistent with lymphoma, at least one lesion with mass effect on brain scan, no response to toxoplasma treatment clinically and by scan	Histology
Lymphoma, non-Hodgkin B cell	None	Histology
lymphoma, Hodgkin	None	Histology
**Neurologic**		
HIV encephalopathy	Cognitive or motor function interfering with usual activity, progressive over weeks or months in the absence of another condition to explain the findings. Should have a brain scan ± CSF examination to exclude other causes.	None
**Other**		
Indeterminate cerebral lesion (s)	Neurologic illness, with evidence for an intracerebral lesion by brain scan, where the differential diagnosis is either cerebral toxoplasmosis. PML, cerebral lymphoma, or HIV encephalopathy	

Based on 1993 Revised CDC classification system (MMWR 1992; 41(RR-17): 1-19) and modified for this trial.

- Visual deficit (at week 10)
- IRIS
- Increased intracranial pressure (clinical or measured)

Uniform management of patients and recording of data will be ensured by the local study staff, who will do clinical assessments daily while admitted and at follow-up visits.

Disability and mortality at 6 months and evidence of morbidity (including IRIS) since last seen (week 10) will be collected with either a structured telephone interview or an outpatient visit. Outpatient visits may occur in the patient’s home when the patient cannot come to the hospital.

### Laboratory monitoring

Inpatient laboratory monitoring will be as shown in the study schedule (section Treatment of CM).

Other investigations may be performed as clinically indicated. Data for the following will be recorded when performed for routine clinical care:

- CSF, if neurologic deterioration (Gram stain and routine culture, ZN stain and mycobacterial culture, India ink stain, and fungal quantitative culture)
- Sputum, if symptomatic (routine culture, ZN stain)
- Urine culture, if urinary symptoms (urine culture)
- Stool examination, if prolonged diarrhoea (microscopy, culture, and parasites)
- Blood cultures, if persistent fever
- Lymph node aspiration (routine and mycobacterial cultures)
- Blood glucose will be measured when CSF is examined or if hyperglycaemia is suspected

A window period of ±2 days outside of the scheduled laboratory tests will apply to all tests that are not baseline inclusion/exclusion criteria.

### Imaging

A chest radiograph must be performed on study entry if it has not been done at the time of diagnosis. The result will be recorded in the CRF. Brain imaging is not mandated by the study. The decision to perform brain imaging will be according to local practice. Results of brain imaging, when available, will be recorded.

### Management of adverse events

Possible side effects of dexamethsone were described earlier.

Given the experience from tuberculous meningitis, gastrointestinal haemorrhage is extremely unlikely (it occurred more frequently in placebo recipients than in patients receiving dexamethasone in the large randomised controlled trial from Vietnam). Gastrointestinal haemorrhage will be treated as per local practices, with cardiovascular support and proton pump inhibitors or H_2_ antagonists. Hyperglycaemia will be managed with insulin.

### Stopping study drug and unblinding rules

The purpose of the randomised, double-blind design of the trial is to protect the results from the potential influence of study staff or patient bias about which treatment is the most effective.

Occasionally it can be necessary to STOP the study intervention (placebo or dexamethasone) or to UNBLIND the patient’s treatment allocation.

*Note:*

- *STOPPING* study drug does not necessitate *UNBLINDING* the treatment allocation. *UNBLINDING* treatment is not necessarily an indication to *STOP* treatment.
- STOPPING or UNBLINDING a patient’s treatment does not mean that they have withdrawn from the study. Patients continue in the study and follow the protocol schedule for visits and investigations until its conclusion at 6 months of follow-up.

#### Stopping study drug

**Figure 4:**
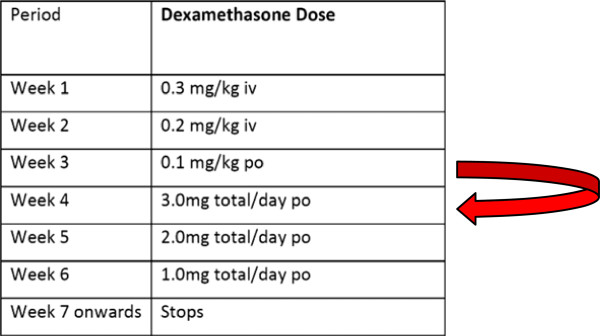
**Dose tapering in the event of stopping study drug.**

Because of the risk of corticosteroid-induced adrenal suppression, the study drug must be stopped in a carefully controlled manner. Few good-quality data are available to enable prediction of the degree of adrenal suppression that occurs with corticosteroid therapy. Therefore, conventional rules for management of corticosteroid withdrawal will be followed for study patients. Tapering the dose of corticosteroids reduces the risk of adrenal suppression. Study drug should only be stopped immediately in two circumstances:

1. if the patient has received four or fewer doses of the study drug, or
2. if the study drug is being replaced by other corticosteroid therapyIn all other circumstances, study drug will be stopped by using dose tapering by moving the patient’s treatment forward on the dosing schedule, as illustrated below: (for example, if the patient is in week 3 of treatment, he or she should be moved immediately onto the week 4 dose, and then continue treatment as per the dose-tapering schedule with weekly reductions in the treatment dose) (Figure 4).

Any changes in patient’s medication must be recorded on the CRF and on the concomitant medication form.

#### Indications for stopping study drug/placebo

The main indication to stop the study drug is if the patient develops a condition for which there is good evidence that steroids are beneficial and should be prescribed. Examples of this are as follows:

1. **Tuberculous meningitis:** Dexamethasone has been demonstrated in a large randomised controlled trial to reduce the risk of death [10]. If a patient develops TB meningitis, the study drug should be stopped, and the patient should receive the appropriate dosage of steroids according to the severity of the disease. This does not necessitate unblinding of treatment allocation.
2. **Intracranial space-occupying lesion with mass effect:** For example, a primary brain tumour such as CNS lymphoma. Space-occupying lesion with mass effect is generally considered to be an indication for steroid therapy. A typical dose would be dexamethasone, 4 mg, 4 times daily. The study drug should be stopped in all patients, and the patient should receive corticosteroid treatment as per local guidelines. This does not necessitate unblinding of treatment allocation.
  **Note**: a suspected cryptococcoma is not an indication to alter the study drug; we do not know whether dexamethasone is indicated in this case, and the study is testing this indication.
3. **Severe pneumocystis pneumonia (PCP):** All patients in the study must receive PCP prophylaxis with co-trimoxazole; this reduces the risk of developing PCP. However, PCP may still occur. Microbiologic confirmation of PCP is not always possible, and the diagnosis is often made on clinical grounds. The chest radiograph typically shows bilateral interstitial, often perihilar, shadowing. The differential diagnosis of this appearance includes bacterial pneumonia and acute respiratory distress syndrome (ARDS) due to cryptococcosis. The gold standard for diagnosis of PCP is to demonstrate the presence of *Pneumocystis jirovecii* in bronchoalveolar lavage washings or in induced sputum samples. Severe PCP is confirmed when there is a typical clinical syndrome is present AND the arterial blood oxygen partial pressure (pO_2_) is less than 70 mm Hg (FiO_2_ ≥ 20%). Prednisolone (40 mg twice daily for 5 days, 40 mg once daily for 5 days, followed by 20 mg/day for 11 days), in combination with high dose co-trimoxazole, has been shown to reduce the risk of death from severe PCP [41]. Where arterial blood gas measurement is not available, diagnosis of severe PCP should be made in accordance with local guidelines. In any patient in whom the diagnosis of severe PCP is made, the study drug must be stopped immediately, and the patient treated with prednisolone in conventional doses, and high dose co-trimoxazole as per local guidelines. This does not necessitate unblinding of treatment allocation.

Any changes to study medication will be recorded in the case-record form.

#### Unblinding

Unblinding means revealing the identity of the study treatment (that is, dexamethasone or placebo). Treatment allocation should be unblinded only if knowing the treatment that a patient has been allocated will result in a change in the patient’s management. The need to unblind is likely to be rare during the study. Unblinding a patient’s treatment allocation does not enable us to tell if a particular adverse event is due to the investigational agent; that conclusion can be drawn only with an analysis of all available data, either at an interim safety analysis (which is done by the independent data and safety-monitoring board) or after the final analysis of the study.

#### Process for unblinding

The decision whether to unblind should be discussed with the lead country investigator or the principal investigator, when possible. Unblinded treatment allocation should be held securely at each site and be available at all times. The responsibility to approve unblinding will be assigned to dedicated staff at each site. Access to treatment allocation should be given only with the approval of one of these dedicated staff. Unblinding will be documented in the case-record form.

### Data on concomitant medications

At each visit, information on other medications, including start dates and reason for taking them, will be documented in the case-record forms.

### Withdrawal from the trial

Patients may voluntarily withdraw from the trial for any reason. If this occurs, the patient will be referred to standard clinical care facilities. The patient’s withdrawal from the trial will not affect the access to the best standard of care within the local health system. With the agreement of the patient, clinical and laboratory assessment should be performed and recorded at the time of withdrawal. Patients may also decide to stop study treatment without withdrawing from the study, in which case, treatment will be adjusted as described later, and follow-up will continue as per the study schedule.

Provided no more than 4 days of study drug have been administered, if a patient wishes to withdraw, the study drug can be stopped immediately. If a patient has received 5 or more days of study drug, then it is possible that adrenal suppression has occurred, and the dose of dexamethasone should be tapered. Thus, if a patient wants to withdraw from the study, and has received 5 or more days of study drug, then the withdrawal phase of steroid dosing will be started immediately; the patient will be switched to the next dexamethasone dose in the steroid-dosing sequence. For example, if on day 16, a patient wants to leave the study, then he or she will be immediately switched to the next phase of steroid/placebo dose (3 mg/day), and the treatment tailed off according to the treatment schedule (Table 4). This continuation of treatment after withdrawal will be fully explained in the informed-consent form. Follow-up will be according to clinical need. All patients in the study will be provided with steroid information cards in their own language. These will detail the need to reduce the dose gradually if they are to be stopped, and to inform other health care workers that they are taking dexamethasone, should they require treatment from a health care worker not involved with the trial. If the patient has an unscheduled period off treatment or not in follow-up, this should be recorded in the case-report forms.

## Definition and assessment of adverse events

### Definition of adverse events

An adverse event (AE) is any undesirable event that occurs to a study participant during the course of the study, regardless of whether that event is considered related to the study drug. An AE can, therefore, be any unfavourable and unintended sign (including an abnormal laboratory finding, for example), symptom, or disease temporally associated with the study drug, whether or not considered related to the study drug.

Examples include the following:

- An increase in severity or frequency of a preexisting abnormality or disorder (events that are marked by a change from the participant’s baseline/entry status)
- All reactions from sensitivity or toxicity to study drug
- Injuries or accidents (for example, for a fall secondary to dizziness, “dizziness” is the event and the injury secondary to the fall is the “outcome”)
- New clinically significant abnormalities in clinical laboratory values, physiological testing, or physical examination.

Stable chronic conditions, such as arthritis, which are present before clinical trial entry and do not worsen are not considered AEs and will be documented in the subject’s clinical chart as medical history.

Clinical or laboratory events are considered adverse events only if they occur after the first dose of study treatment and before the patient completes trial participation. (See later for reporting of adverse events.)

### Definition of serious adverse events

An AE is considered to be "serious" if it results in one of the following outcomes

- Death,
- Life-threatening event (the subject was at immediate risk of death at the time of the event; it does not refer to an event that hypothetically might have caused death if it were more severe),
- Inpatient hospitalisation or prolongation of existing hospitalisation
- Persistent or significant disability/incapacity (a substantial disruption of a person's ability to conduct normal life functions),
- Congenital anomaly/birth defect,
- Important medical event that may not be immediately life-threatening or result in death or hospitalisation but may jeopardise the patient or may require intervention to prevent one of the other outcomes listed in the earlier definition.

### Definition of unexpected serious adverse events

Unexpected serious adverse events are untoward medical events that fit one or more of the previously explained criteria for SAEs and that are not considered a part of normal clinical progression of disease or an expected drug reaction. Any event that becomes of concern to the investigators or study doctors during the course of the trial may be reported as a USAE.

### Assessment of adverse events

Adverse events will be defined according to the Common Terminology Criteria for Adverse Events (CTCAE) definitions: http://ctep.cancer.gov/protocolDevelopment/electronic_applications/ctc.htm. New AIDS-defining events will be defined according to the revised CDC criteria modified for this trial (see Table 5, and Appendix 6). In the event that an adverse event is not described within the CTCAE definitions, or a new AIDS-defining event, as defined in Table 5, the following generic severity grading will be used:

**Grade 1 Mild;** asymptomatic or mild symptoms; clinical or diagnostic observations only; intervention not indicated.

**Grade 2 Moderate;** minimal, local, or noninvasive intervention indicated; limiting age-appropriate instrumental Activities of Daily Living (ADLs)*.

**Grade 3 Severe or medically significant but not immediately life-threatening;** hospitalisation or prolongation of hospitalisation indicated; disabling; limiting self-care ADL**.

**Grade 4 Life-threatening consequences;** urgent intervention indicated.

*Instrumental ADLs refer to preparing meals, shopping for groceries or clothes, using the telephone, managing money, and so on.

**Self-care ADLs refer to bathing, dressing and undressing, feeding self, using the toilet, taking medications, and not bedridden.

Details on the grading of specific adverse events can be found in Appendix 6. [Note: “Life-threatening” as a severity grade is not necessarily the same as “life-threatening” as a “serious” criterion used to define a serious adverse event. The former is a “potential” threat to life, and the latter is an “immediate” threat to life.]

A **laboratory abnormality** must be recorded as a clinical adverse event only if it is associated with an intervention. Intervention includes, but is not limited to, discontinuation of a current treatment, dose reduction/delay of a current treatment, or initiation of a specific treatment. In addition, any medically important laboratory abnormality may be reported as an adverse event at the discretion of the investigator. This would include a laboratory result for which no intervention is needed, but the abnormal value suggests a disease or organ toxicity. Laboratory events will be graded according to CTCAE definitions.

If clinical sequelae are associated with a laboratory abnormality, the diagnosis or medical condition should be reported as the adverse event (for example, renal failure, haematuria), not the laboratory abnormality (for example, elevated creatinine, urine RBC increase).

## Recording and reporting of adverse events and protocol violations

### Adverse event recording

Grade 3 and 4 adverse events will be recorded in the case report form and entered into the study database. Grade 1 and 2 events will not be recorded, because in a severe disease such as CM, the number of low-grade adverse events is likely to be high, representing a large reporting burden, which may affect the quality of recording and reporting of more important grade 3 and 4 adverse events.

### Adverse event reporting

Serious adverse events and serious unexpected adverse events will be **reported** to the Principal Investigator within 7 days of occurrence, or sooner, according to local requirements. The Principal Investigator will report all unexpected serious adverse events to the DMEC within 10 days of occurrence. Unexpected serious adverse events will be reported to the responsible ethical committees within 10 days of occurrence, or as required by the committee.

### Protocol violations

Protocol violations are events that contradict or omit protocol instructions. Violations that compromise patient safety or the integrity of trial data will be recorded and reported to the responsible Ethics Committees, as required by the regulations of each Committee.

## Statistics

### Sample size and power considerations

The trial is powered for the primary end point (that is, overall survival during the 10-week follow-up period).

Few data exist on which to estimate the potential effect size of dexamethasone on mortality from CM. We have based our estimate of the effect size on data from tuberculous meningitis, which shares clinicopathologic features with CM. In a large RCT of dexamethasone to treat tuberculous meningitis in Vietnam, the hazard ratio (HR) for death was 0.69 at 9 months in favour of dexamethasone [25]. Most of this effect occurred during the first 3 months of treatment. The dose of dexamethasone we will test is the same as that used in that trial. Additionally, in a study of dexamethasone in bacterial meningitis in Vietnam, the HR was 0.43 in patients with microbiologically confirmed disease, and 0.59 in a study based in Europe, again in favour of dexamethasone [34, 35]. These analyses were all based on the intention-to-treat principle (including noncompliant patients), and it seems sensible to expect a similar rate of noncompliance in our study. Therefore, we believe a target hazard ratio of 0.7 in favour of dexamethasone to be reasonable for our trial.

To detect such a risk reduction of 30% with 80% power at the two-sided 5% significance level, a total of at least 247 deaths must be observed. Based on historical data and consultation with principal investigators from all study sites, the 10-week mortality risk in the control group is expected to be between 30% and 50% [38], and the target hazard ratio of 0.7 corresponds to absolute risk reductions in mortality from 30% to 22%, or from 50% to 38%. Assuming an overall 10-week mortality of at least 30% in our study population, recruitment of 824 patients is sufficient to observe the target number of deaths. Allowing for some loss to follow-up, the total sample size for this study is 880 patients.

A major aim of this trial is to generate robust evidence across both continents. To achieve this goal, we aim to recruit roughly similar numbers of patients from each continent.

Event-driven stopping of the trial after 247 observed deaths is not foreseen even if this should occur prior to recruiting 880 patients. Thus, the study will be conservatively powered if the study mortality is larger than 30%. For example, if overall mortality was around 50% and 412 deaths were observed during the study, power would increase to 95% for a true hazard ratio of 0.7 and to 83% for a true hazard ratio of 0.75.

### Analysis

#### Analysis of the primary end point and 6-month mortality

The analysis will be based on a stratified Cox proportional hazards model allowing for separate baseline hazards for each continent (Asia or Africa) and treatment allocation as the only covariate. The stratification is based upon the expectation of different mortalities in the control arm by continent but similar (relative) effects of the intervention across continents. The proposed test is essentially equivalent to using a stratified log‒rank test to compare the two treatment arms. We prefer to use the Cox model as it automatically provides treatment effect estimates and confidence intervals in addition to the *P* value.

In a second stage, overall survival will be modelled by using the Cox proportional hazards regression model with stratification by continent and the following covariates (in addition to the treatment group): country, baseline fungal load, Glasgow coma score less than 15, and ARV status at study entry (ARV naïve or experienced).

Potential heterogeneity of the treatment effect will be assessed based on appropriate interaction (likelihood ratio) tests and the following predefined subgrouping variables:

- Continent
- Country
- IDSA indications for steroid treatment at baseline (cryptococcoma with mass effect, acute respiratory distress syndrome or IRIS: yes or no)
- Glasgow coma score <15 (yes or no)
- Naïve to ARVs at study entry versus on ARVS at study entry

All Cox regressions and sub-group analyses will be performed for the primary end point (10-week survival) as well as for 6-month survival. Kaplan-Meier plots and explicit survival estimates at 10 weeks and 6 months of follow-up will also be calculated for the full populations and for each continent separately.

#### Analysis of secondary end points

##### Neurologic disability

The disability score at week 10 and month 6 of follow-up is defined as the higher (worse) of the “simple question” and the Rankin score assessed at that time point and will be categorised as good outcome, intermediate disability, severe disability, or death (in case the patient died before the respective time point) as previously described [10] (also see Table 1). The proportion of patients with a good outcome will be compared between the two arms with a logistic regression adjusted for continent (in addition to the treatment arm). Patients lost to follow-up will be analysed according to their last recorded disability status. If the rate of patients lost to follow-up exceeds 10%, we will also perform an alternative analysis based on multiple imputation of missing values.

##### Rate of CSF sterilisation during the first 2 weeks (based on available data from selected sites only)

Fungal decline in the first 14 days will be modelled with a joint model for longitudinal and survival data. The longitudinal part of the model will be a linear mixed-effects model with longitudinal log-CSF quantitative culture fungal counts as the outcome, continent and interaction terms between the treatment groups and the time since enrolment of the measurement as fixed covariates, and a random patient-specific intercept and slope. The survival part of the joint model models mortality up to 2 weeks depending on the treatment group, continent, and the patient-specific random intercepts and slopes. The survival part acts as a missing data mechanism to allow potentially informative truncation of quantitative count measurements due to death.

Longitudinal measurements of intracranial pressure during the first 2 weeks will be modelled in the same way.

##### Adverse events

The frequency of serious and grade 3 and 4 adverse reactions as well as the frequency of specific adverse events will be summarised (both in terms of the total number of events as well as the number of patients with at least one event). The proportion of patients with at least one such event (overall and for each specific event separately) will be summarised and (informally) compared between the two treatment groups based on the Fisher Exact test.

**The rate of IRIS and the rate of relapse** (defined as antifungal treatment intensification or re-treatment) will be modelled with cause-specific proportional hazards models stratified by continent, taking into account the competing risk of prior death. Cumulative incidence functions for the competing events will also be calculated and displayed by treatment arm. Secondary time-to-event end points (time to new AIDS-defining illness or death and time to new neurologic event or death) will be analysed with Kaplan-Meier curves and Cox regression models, as described for the primary end point expressed earlier. The proportion of patients with blindness or visual deficit will be compared between the two arms with a logistic regression (as for the proportion of patients with good disability described earlier).

#### Analysis populations

The primary analysis population for all analysis is the full analysis population containing all randomised patients except for those mistakenly randomised without CM. Patients will be analysed according to their randomised arm (intention-to-treat). In addition, the primary end point will be analysed on the per-protocol population, which will exclude the following patients: major protocol violations and those receiving less than 1 week of administration of the randomised study drug for reasons other than death.

## Interim analysis and role of the Data Monitoring and Ethical Committee (DMEC)

An independent DMEC will oversee the trial. Unexpected serious adverse events with treatment allocation blinded will be reported to the DMEC within 10 days of occurrence and followed up until resolution. The DMEC will perform formal interim analyses after every 50 deaths. According to the sample-size calculations, we expect to observe around 247 deaths during the course of the study. Thus, four to five planned formal interim analyses after 50, 100, 150, 200, and (possibly) 250 deaths will take place.

At these interim analyses, the DMEC will receive a report including unblinded summaries of mortality, serious adverse events, grade 3 and 4 adverse events, and estimates of the rate of CSF sterilisation during the first 14 days (from selected sites only) by treatment arm. The report will be prepared by the DMEC statistician and distributed to all DMEC members for review. Based on these data, the committee will make recommendations on the continuation, cessation or amendment of the study. The study statistician will remain blinded throughout the study but will aid in setting up the code for generating the interim analysis summaries. The randomisation list will be sent to the DMEC statistician directly from the study central pharmacist.

Stopping for efficacy of dexamethasone at an interim analysis is foreseen only if the benefit of adjuvant treatment with dexamethasone is shown “beyond reasonable doubt.” The Haybittle-Peto boundary, requiring *P* <0.001 at interim analysis to consider stopping for efficacy, will be used as guidance. Stopping for harm of dexamethasone should be considered if an unfavourable trend emerges, sufficiently large to rule out a clinically relevant benefit of dexamethasone. Conclusive evidence that dexamethasone is harmful is not sought, as continued exposure of patients to a nonbeneficial novel treatment that could be harmful appears to be unethical. To support the DMEC decision, the DMEC will receive conditional power curves in addition to the summaries described earlier. Conditional power assesses the probability that a benefit of dexamethasone will eventually be detected, conditional on the data accrued so far. If this probability is low for a wide range of reasonable assumed treatment effects (including the target hazard ratio of 0.7 from the original sample-size calculation), this suggests little reason to continue the trial because the treatment is unlikely to show benefit.

Importantly, the DMEC recommendations will not be based purely on statistical tables but will also use clinical judgment.

As the dissemination of preliminary summary data could influence the further conduct of the trial and introduce bias, access to interim data and results will be confidential and strictly limited to the involved independent statistician and the monitoring board, and results (except for the recommendation) will not be communicated to the outside and/or clinical investigators involved in the trial.

Further reviews will be at the discretion of the DMEC or the request of the Trial Steering Committee. All DMEC reports, replies, or decisions will be sent to the Trial Steering Committee and the responsible Research Ethical Committees.

## Ethical considerations

### Declaration of Helsinki and good clinical practice

The Investigator will ensure that this study is conducted in compliance with the current revision of the Declaration of Helsinki (Seoul 2008) and the Medical Research Council Guidelines on Good Clinical Practice (1998).

### Ethical review

The Oxford University Tropical Research Ethics Committee (OxTREC) is the ethical committee of reference for this trial. The study protocol and its associated documents were submitted to OxTREC and all other ethical committees, as required by local regulation at each site. Ethical approval has been obtained from:

- OxTREC, Oxford, UK 25-12
- Ministry of Health, Vietnam
- Hospital for Tropical Diseases, Ho Chi Minh City, Vietnam
- Ministry of Health, Laos 039/NECHR
- Faculty of Medicine, Universitas Indonesia, Indonesia 623/H2.F1/ETIK/2012
- Mahidol University, Thailand MUTM 2012-051-01
- Institute for the Development of Human Research Protections,
- Thailand 04CN
- Uganda Council for Science and Technology HS1264
- National Drug Authority, Uganda 020/ESR/NDA/DID-01/2013
- Ministry of Health, Malawi #1077
- Pharmacy, Medicines and Poisons Board, Malawi PMPB/CTRC/111/3105201354
- University of Toronto, Canada 28199

The principal investigator will submit and obtain approval where necessary from the ethical committee of reference and the responsible local committees for all substantial amendments to the original approved documents. Annual reviews of the trial will be conducted by the ethical committee of reference and as required by all other committees.

### Informed consent

The study staff will discuss the study with all potential adult participants or, in the case of a participant who is unable to give informed consent independently, with a representative appropriate within the local culture. Study staff will describe the purpose of the study, the study procedures, possible risks/benefits, the rights and responsibilities of participants, and alternatives to enrolment. The participant or representative will be invited to ask questions that will be answered by study staff, and they will be provided with appropriate numbers to contact if they have any questions subsequently. If the participants or representatives agrees to participate, they will be asked to sign and date an informed consent form. A copy of the form will be given to them to keep. If required, the participant or representative will be given up to 24 hours to consider the study, provided the participant remains eligible for the study.

Participants whose consent was given by a representative will be approached to consider consent independently, if at any time during study participation he or she becomes able to consider consent independently.

In addition to these procedures, illiterate signatories will have the informed-consent form read to them in the presence of a witness who will sign to confirm that the form was read accurately and that the participant or representative agrees to participation. All patient-information sheets and Consent/Assent forms will be written in the local language and will use terms that are easily understandable. Clinical care will not be delayed in any case during consideration of consent.

### Risks

This study will use a drug that has been studied thoroughly and its toxicities are well described. Details can be found in the Study Treatment section of this protocol. Patients will be closely monitored for all adverse events and treated as per standard of care. Additional volumes of blood and cerebrospinal fluid will be taken for research tests. These volumes have very little risk of affecting the participant’s health. Some phlebotomy may be performed more often than is required by clinical care. This procedure carries the small risk of bruising and infection.

Dexamethasone may be growth suppressing in children and fetuses. Therefore, children and pregnant women have been excluded from this trial.

### Benefits

It is unknown whether study participants who receive study treatment will benefit. The additional monitoring and follow-up of patients by dedicated study staff may be of benefit to patients treated in resource-limited settings. Treatment costs for trial participants will be supported, including payment of all study treatment and standard antifungal treatment.

Training in laboratory and clinical procedures, research methods, and good clinical practice, will be given to all participating centres. Investigators will engage with the HIV/AIDS community in each setting to ensure that trial conduct is cohesive with local patient-support services.

### Alternatives to study participation

The alternative to participation in this study is routine care by the doctors in the hospital. Patients will be responsible for their own treatment costs as per local norms and hospital policy.

### Confidentiality

Participants will be assured that all information generated in this study will remain confidential. The trial staff will ensure that the participants’ anonymity is maintained. Participant’s names will be recorded at the time of enrolment to allow their identification at follow-up visits. However, identifiable information will be linked to stored data or samples only by a protected Master List. This list will not be shared outside the study staff. All documents will be stored securely, and all reports or samples will be coded without identifying information. Direct access will be granted to authorised representatives from the host institution and the regulatory authorities, if applicable, to permit trial-related monitoring and inspections.

### Withdrawal of participants from the study

Each participant has the right to withdraw from the study at any time. All patients who withdraw will be referred for treatment as per routine clinical care. The reason for withdrawal will be recorded in the CRF. Study drug will be managed as detailed under Stopping Study Drug.

### Sample sharing and storage

Samples collected will be used for the purpose of this study as stated in the protocol and stored for future use in studies not yet conceived, which may include genetic studies. Consent will be obtained from subjects for genetic testing and for sample storage and/or shipment of specific samples to collaborating institutions for investigations that cannot be performed locally. Any proposed plans to use samples other than for those investigations detailed in this protocol will be submitted to the relevant ethics committees before any testing.

The participants will be identified only by a study-specific participant number and/or code in any database. The name and any other identifying detail will not be included in any study data electronic file.

### Sponsorship and insurance

The University of Oxford has appropriate insurance-related arrangements in place in respect of the University's role as research sponsor for this study.

## Data

### Data collection and entry

Source documents will be generated during the study by the site study staff at participating institutions. Source documents include all original recordings of observations or notations of clinical activities, and all reports and records necessary for the evaluation and reconstruction of the clinical trial. Source documents include, but are not limited to, the subject’s medical records, research case record forms (paper or electronic), laboratory reports, ECG tracings, x-rays, radiologist’s reports, subject’s diaries and questionnaires, biopsy reports, ultrasound photographs, progress notes, pharmacy records, and any other similar reports or records of procedures performed during the subject’s participation in the study.

Access to applicable source documents is required for study purposes. The site investigators are responsible for maintaining any source documentation related to the study. Source documentation should support the data collected on the CRF when the CRF is not the original site of recording. Source documentation must be available for review or audit by the sponsor or designee and any applicable regulatory authorities.

Case Report Forms (CRFs) will be used as a data collection tool. The study team will transfer the information from the source documents onto the CRFs. CRFs may be used as source documents if they are the primary data-collection tool for specified data, as documented in written standard operating procedures. The site Investigators are responsible for maintaining accurate, complete, and up-to-date records. These forms are to be completed on an ongoing basis during the course of the study by authorised individuals.

Corrections to paper CRFs must be initialled and dated by the person making the correction and must not obliterate the original entry. All CRFs should be reviewed by the designated study staff and signed as required with written or electronic signature, as appropriate.

Selected study members (study doctors or nurses) will be trained on how to enter all clinical data as source information from the CRFs and from laboratory source documents into an Internet-based computerised data-entry system called CliRes hosted by OUCRU Viet Nam. Data entry will occur simultaneously, as data are being generated during the trial as soon as possible after the information is generated. Data may be manually entered or scanned and electronically uploaded, dependent on available software. Source documents and electronic data will be verified according to the Trial Monitoring Plan.

After study completion and the main analyses and publication of the study results, the study sub-datasets consisting of the patient data from particular recruiting sites will be available to the investigators from those sites.

### Record retention

The investigator is responsible for retaining all essential documents listed in the MRC guidelines on Good Clinical Practice. All essential documentation for all study subjects is to be maintained in original paper format by the investigators in a secure storage facility for a minimum of 3 years and as required by local regulations thereafter. All essential documentation will be converted from paper to electronic format (if required) and stored centrally for at least 10 years after the completion of the trial and as required by local regulations thereafter. All stored records are to be kept secure and confidential.

## Monitoring

The trial will be conducted in compliance with this protocol, Medical Research Council Guidelines of Good Clinical Practice, and any applicable regulatory requirement(s).

The study will be adequately monitored by the OUCRU or their designate. Monitors will visit the clinical research site to monitor all aspects of the study in accordance with the appropriate regulations and the approved protocol. The objectives of a monitoring visit will be (a) to verify the existence of adequately signed informed-consent documents for each enrolled subject; (b) to verify the prompt, complete, and accurate recording of all monitored data points, and prompt reporting of all SAEs and unexpected SAEs; (c) to compare abstracted information with individual subjects’ records and source documents (subjects’ charts, case report forms, laboratory analyses and test results, physicians’ progress notes, nurses’ notes, and any other relevant original subject information); (d) to verify the supply and condition of the study drug and the accurate and secure assignment of randomisation code; and (e) to ensure protection of study subjects, investigators’ compliance with the protocol, and completeness and accuracy of study records. The monitors also will inspect the clinical site regulatory files to ensure that regulatory requirements and applicable guidelines are being followed. During the monitoring visits, the investigator (and/or designee) and other study personnel will be available to discuss the study progress and monitoring visit.

## Discussion

The trial commenced recruiting patients in February 2013 and by the end of May 2014 had recruited 357 patients. Two interim analyses were made by an independent data monitoring and ethics committee, as predetermined, and on both occasions, it was concluded that the trial was being conducted well, and should continue. An investigators’ meeting was held in Vietnam to develop the collaborative network, share best-practice qualities, discuss recruitment strategies, and consider opportunities for substudies.

Initially, the trial was designed to recruit 550 patients from Asia and 330 from Africa (880 total) with the rationale being that the 10-week death rate in patients in Vietnam would be 30%, and in Malawi, 50% [38], and that the target hazard ratio of 0.7 corresponded to an absolute risk reduction in mortality from 30% to 22%, or from 50% to 38%. These national mortality rates were assumed to be representative at the continental level, and recruitment of 500 patients from Asia and 300 patients from Africa (800 patients in total) would have been sufficient to observe the target number of deaths, a total of 880, allowing 10% loss to follow-up.

As the trial progressed, it became apparent that this strict division could hamper recruitment and was based on information that suggested a larger difference between Asian and African patients than was being observed in the blinded data. After discussions amongst the investigators and the trial steering committee, this requirement was replaced by a more-lenient statement that the study would aim to recruit roughly similar numbers of patients from each continent.

## Publication

Any publication or presentation during the active phase of the study must have permission from the Investigators. The investigators will define the strategy for publication, resolve any problems of authorship, and maintain the quality of publications. All publications will acknowledge the appropriate authors and funding sources according to normal academic practice. The investigators are the custodians of the data and specimens generated from this trial.

## Trial status

In total, 417 of 880 patients have been recruited by 6 August, 2014.

## Appendix 1 Cryptococcal quantitative cultures, cryptococcal archiving: standard operating procedure

### Aim

To describe how to safely undertake quantitative culture of CSF for Cryptococci and how to store isolates as part of the Cryptodex study (see Table 6 for safety considerations).

**Table 6:** **COSHH risk assessment - University of Oxford COSHH assessment form**

**Description of procedure**	**Substances used**
Semi-quantitative culture of CSF for HIV and storage of isolates	Sabouraud’s agar
	CSF
	*Cryptococcus* sp.
**Quantities used**	**Frequency of use**
Up to 8 ml CSF	Daily
**Hazards identified**	**Could a less hazardous substance be used instead?**
1. Blood-borne viruses: CSF samples for this study will be from patients with HIV and possibly other blood-borne viruses.	No
2. *Cryptococcus neoformans* var *grubii* and *Cryptococcus gattii* are ACDP category 2 organisms. They pose a small risk of infection to workers, treatment is available, and there is no risk of person to person transmission.	
**What measures have you taken to control risk?** A laboratory coat, gloves and goggles or glasses must be worn at all times when working on specimens. All patient samples and cultures should be handled within a Class II biological safety cabinet. All staff must be checked for hepatitis B virus immunity before commencing work as per local procedures, and vaccinated as appropriate. In the event of a splash or other injury, work will be stopped immediately, and local guidelines will; be followed.	
**Checks on control measures**	
Observation and supervision by senior staff.	
**Is health surveillance required?**	**Training requirements:**
No	All staff to be trained in the above SOP prior to use.
**Emergency procedures**:	**Waste disposal procedures**:
Local guidelines for splashes and inoculation injuries.	All waste will be autoclaved prior to disposal.

### Principle

Serial 10-fold dilutions of CSF are made, and a known volume is cultured on Sabouraud agar. After incubation, the number of colonies growing is counted and used to calculate the approximate numbers of *Cryptococcus* sp. in the original sample. Initial isolates and any variants, including those that might represent drug-resistant strains, are stored by using a commercial system.

### Equipment

1. Volumetric pipettes capable of delivering 100 μl and 900 μl volumes
2. Sterile tips for these
3. Bottles containing 5 to 10 ml sterile water
4. Lidded plastic tubes for 1-ml volumes of CSF dilutions (four per sample)
5. Sabouraud dextrose agar plates (five per sample)
6. Sterile 1.5 ml Eppendorf tubes for storing CSF
7. Storage boxes (Nalgene or other)
8. Pro-Lab Microbank Beads or :Protect” tubes for archiving isolates
9. Safety cabinet (Class II)
10. Vortex

### Method

1. **Plate preparation**
  1.1 Dry the Sabouraud plates in an incubator (30°C) for 30 minutes before use.
  1.2 Mark each plate into two halves by using a permanent marker.
  1.3 Label each plate with
    - the trial study number (if this is not yet known, use the patient's name and date of birth or age),
    - the date the sample was taken,
    - the dilution: that is, Neat, 10^-1^, 10^-2^, 10^-3^, 10^-4^
2. **Dilution preparation**
  2.1 Dispense 900 μl of sterile distilled water aseptically into each tube in the Class II BSC, and fasten the lid tightly.
  2.2 Label each tube with the Study number and the dilution (10^-1^, 10^-2^, 10^-3^, and 10^-4^).
3. **Samples**
  3.1 Samples should be processed as soon as possible after being taken. If any delay occurs, samples should be refrigerated at 4° to 8°C.
  3.2 All processing should take place in a Class II BSC.
  3.3 Neat and diluted samples should be vortexed before every step to ensure even mixing (that is, before making the next serial dilution and before inoculating the plate).
  3.4 Using the volumetric pipette and a fresh sterile tip, take 100 μl of vortexed CSF and add it to the tube marked 10^-1^.
  3.5 Vortex the 10^-1^ dilution, remove 100 μl of this dilution by using a fresh sterile tip and add it to the tube marked 10^-2^.
  3.6 Vortex the 10^-2^ dilution, remove 100 μl of this dilution by using a fresh sterile tip, and add it to the tube marked 10^-3^.
  3.7 Vortex the 10^-3^ dilution, remove 100 μl of this dilution by using a fresh sterile tip, and add it to the tube marked 10^-4^.
4. **Plate inoculation**
  4.1 Plates should be inoculated immediately after making the serial dilutions.
  4.2 Use the plates labelled as in section Study design.
  4.3 Vortex the 10^-4^ dilution, take a fresh sterile pipette tip, and immediately remove 100 μl and use it to inoculate half the plate labelled 10^-4^ by dispensing approximately 20 drops of 5 μl.
  4.4 Repeat 4.3 for the other half of the plate.
  4.5 Vortex the 10^-3^ dilution, take a fresh sterile pipette tip, and immediately remove 100 μl and use it to inoculate half the plate labelled 10^-3^ by dispensing approximately 20 drops of 5 μl.
  4.6 Repeat 4.5 for the other half of the plate.
  4.7 Vortex the 10^-2^ dilution, take a fresh sterile pipette tip, and immediately remove 100 μl and use it to inoculate half the plate labelled 10^-2^ by dispensing approximately 20 drops of 5 μl.
  4.8 Repeat 4.7 for the other half of the plate.
  4.9 Vortex the 10^-1^ dilution, take a fresh sterile pipette tip, and immediately remove 100 μl and use it to inoculate half the plate labelled 10^-1^ by dispensing approximately 20 drops of 5 μl.
  4.10 Repeat 4.9 for the other half of the plate.
  4.11 Vortex the Neat CSF, take a fresh sterile pipette tip, and immediately remove 100 μl and use it to inoculate half the plate labelled Neat by dispensing approximately 20 drops of 5 μl.
  4.12 Repeat 4.11 for the other half of the plate.
  4.13 Incubate all plates at 30°C for 1 week.
5. **Reading samples**
  5.1 Start a new recording sheet for each patient.
  5.2 Record the patient’s study number on each sheet.
  5.3 Read the plates on day 3, day 5, and day 7.
  5.4 Count the colonies on each plate by holding the plate over a dark background.
  5.5 Record plates with confluent growth as ”confluent growth.”

6. **Calculating the results**
  6.1 Use the plate that has between 10 and 100 typical Cryptococcus colonies on each side to perform the count (if in doubt about the identity, do a Gram stain and India ink). If no plate has more than 10 colonies, then use the plate with positive growth to estimate the fungal burden.
  6.2 Count the number of colonies on both sides of this plate, and calculate the average.
  6.3 Calculate the number of cfu/ml of CSF by multiplying the number of colonies by 10 (as only 100 μl was cultured) and then by the dilution factor (that is, 20 colonies on Neat plate =20 × 10 × 10^0^ = 200 cfu/ml
    ○ 20 colonies on 10^-1^ plate =20 × 10 × 10^1^ = 2,000 cfu/ml
    ○ 20 colonies on 10^-2^ plate =20 × 10 × 10^2^ = 20,000 cfu/ml
    ○ 20 colonies on 10^-3^ plate =20 × 10 × 10^3^ = 200,000 cfu/ml
    ○ 20 colonies on 10^-4^ plate =20 × 10 × 10^4^ = 2,000,000 cfu/ml

7. **Archiving samples**
  7.1 The first available C. *neoformans* growth for each patient should be archived by using the locally preferred method (for example, Pro lab Microbank beads, “Protect” tubes or TSB +20% glycerol).
  7.2 Use the Neat sample from Day 1 (or a later day of the study if no Day 1 sample is available).
  7.3 Run the sterile loop through all the colonies from each side of the Neat culture plate and inoculate the beads as per the manufacturer's instructions. Run the loop through all the colonies rather than using a single colony because Cryptococcus populations within patients may be heterogeneous.
  7.4 Repeat for the other side of the plate with a new set of beads (so two sets of each sample are archived).
  7.5 If colonies of different morphology are from the same plate, then archive these separately, and describe them on the tube label (for example, patient XXXX, “smooth colony,” and Patient XXXX “irregular colony.”
  7.6 Label each bead tube with the study number, ”*C. neoformans”* and the date that the sample WAS TAKEN FROM THE PATIENT (not the date it was cultured or archived).
  7.7 Try not to use a plate if it is contaminated with mould; - subculture first if no mould-free plates are available.
  7.8 Most patients will need only the earliest available sample to be archived, but please also archive the isolate from any sample where increasing growth is present throughout the course of treatment (for example, if the day 14 sample has a higher colony count than the day 7, please also archive the day 7 and day 14 colonies).

## Appendix 2 Definition of cryptococcal meningitis-associated immune reconstitution syndrome

### Case definition for paradoxical cryptococcal immune reconstitution inflammatory syndrome in HIV patients

### Antecedent requirements

- Taking antiretroviral therapy
- Cryptococcal disease diagnosed before ARV by positive culture or typical clinical features plus positive India ink staining or antigen detection
- Initial clinical response to antifungal therapy with partial or complete resolution of symptoms or signs, fever, or other lesions, or reduction in CSF cryptococcal antigen concentration or quantitative culture

### Clinical criteria

- Event occurs within 12 months of ARV initiation, reintroduction, or regimen switching after previous failure
- Clinical disease worsening with one of the following inflammatory manifestations of cryptococcosis:
- Meningitis
- Lymphadenopathy
- Intracranial space-occupying lesion or lesions
- Multifocal disease
- Cutaneous or soft-tissue lesions
- Pneumonitis or pulmonary nodules

### Other explanations for clinical deterioration to be excluded

- Non-adherence or suboptimum antifungal therapy, indicated by an increase in quantitative culture or antigen titer, or any positive cryptococcal culture after 3 months of antifungal therapy
- Alternative infection or malignant disease in the affected site
- Failure of ART excluded if possible (for example, failure to achieve ≥1 log10 viral load by 8 weeks of ARV)

ARV, antiretroviral therapy; CSF, cerebrospinal fluid.

## Appendix 3: Management of increased intracranial pressure

Increased intracranial pressure is a frequent complication of cryptococcal meningitis. The mechanism is not clear, but probably is a combination of impaired CSF drainage, increased CSF production, cerebral oedema, and inflammation. Adults have approximately 175 ml of cerebrospinal fluid, and the 24-hour production of CSF is on the order of 550 ml. The normal CSF pressure recorded by lumbar puncture with the patient reclining in the left lateral position is 5 to 18 cm of CSF. Few data guide the management of increased intracranial pressure in patients with cryptococcal meningitis, but the recommendations of the IDSA guidelines are that the CSF pressure should be identified at baseline. If the CSF pressure is ≥25 cm of CSF and symptoms of increased intracranial pressure are present during induction therapy, relieved by CSF drainage (by lumbar puncture, reduced the opening pressure by 50% if it is extremely high or to a normal pressure of ≤20 cm of CSF). If persistent pressure elevation of ≥25 cm of CSF exists and symptoms, repeat lumbar puncture daily until the CSF pressure and symptoms have been stabilised for 1 to 2 days.

Brain imaging should be considered before lumbar puncture in patients with focal neurologic signs or profound coma, although its sensitivity for predicting cerebral herniation is poor [4].

## Appendix 4 Antifungal treatment

All patients will receive amphotericin B deoxycholate 1 mg/kg/day for 2 weeks combined with fluconazole, 800 mg/day, for the first 2 weeks after randomisation. This will be followed by 800 mg fluconazole per day for 8 weeks. After this point, patients are switched to fluconazole, 200 mg/day, secondary prophylaxis. This continues until the patient has had sustained immune reconstitution (CD4 count >100 cells/μl) secondary to antiretroviral therapy. This is consistent with current IDSA and WHO guidelines for the treatment of cryptococcal meningitis.*

*Perfect JR, *et al*: **Clinical** Practice Guidelines for the Management of Cryptococcal Disease. *Clin Infect Dis* 2010, **50**:291–322., and **Rapid Advice: Diagnosis, Prevention and Management of Cryptococcal disease in HIV-infected Adults, Adolescents and Children: World Health Organization**; 2011

## Appendix 5 Amphotericin administration and complications

### Administration

Renal impairment occurs in 80% of patients receiving amphotericin, and is reversible provided the total dose does not exceed 4 g. Evidence suggests that sodium depletion increases the chance of amphotericin-induced nephrotoxicity. Administration of normal saline before amphotericin infusion reduces this risk. This must be followed by a dextrose flush, because amphotericin is incompatible with normal saline.

1. Flush line with 50 to 100 ml dextrose 5%.
2. Administer 1,000 ml normal saline containing 20 mmol potassium chloride over a 2- to 4-hour period (contraindications: fluid overload, cirrhosis, heart failure)
3. Flush line with 50 to 100 ml dextrose 5%
4. Administer amphotericin

Dose: 1 mg/kg/day

Infusion solution: 5% dextrose

Rate of infusion: 4 hours

### Management of amphotericin-induced renal impairment

1. Frequent monitoring in all patients of electrolytes, creatinine and urea.
2. There is no need to reduce the dose of amphotericin unless the creatinine is >3 times upper limit of normal (ULN).
3. If creatinine exceeds 3 times ULN, discontinue amphotericin for 1 day, then reintroduce at half the previous dose, and gradually increase this dose to the target level over the next 2 to 3 days, carefully observing renal function.

### Management of amphotericin-induced hypokalaemia

Reversible hypokalaemia is common in amphotericin treatment. Potassium levels should be checked twice weekly during the period of amphotericin administration. Hypokalaemia can be treated with oral potassium chloride (1 to 2 tablets 2 to 3 times daily according to response). There is evidence that this can be helped by administering a small daily dose of amiloride (10 mg) orally.

### Management of rigors

A minority of patients may develop rigors and fevers when starting their infusions with amphotericin. This symptom usually resolves after a few days, but can be helped by prophylactic chlorphentiramine or aspirin.

Anaphylaxis is rare with amphotericin, occurring in less than 1% of patients.

### References

SH Khoo, J Bond, DW Denning: **Administering amphotericin B: a practical approach**. *J Antimicrobial Chemother* 1994, **33:** 203–221

## Appendix 6 Common terminology criteria for adverse events

Hard copies of the CTCAE will be provided for the study staff. Details of the CTCAE criteria can be found at: http://ctep.cancer.gov/protocolDevelopment/electronic_applications/ctc.htm.

## Abbreviations

ARV: anti-retroviral therapy
DfID: Department for International Development, UK
CM: cryptococcal meningitis
HIV: human immunodeficiency virus
AIDS: acquired immune deficiency syndrome
ADL: activity of daily living
DMEC: Data Monitoring and Ethical Committee
CDC: Centers for Disease Control and Prevention
GCP: good clinical practice
AE: adverse event
ATCG: AIDS Clinical Trials Group
CTCAE: Common Terminology Criteria for Adverse Events
(DB)RCT: (double-blind) randomised control trial
ICER: incremental cost-effectiveness ratio
ICP: intracranial pressure
IRIS: immune reconstitution inflammatory syndrome
IDSA: Infectious Disease Association of America
CSF: cerebrospinal fluid
VEGF: vascular endothelial growth factor
TBM: tuberculous meningitis
MRC: Medical Research Council, UK OI, opportunistic infection
OxTREC: Oxford University Tropical Research Ethics Committee
PCP: *Pneumocystis jirovecii* pneumonia QALY, quality adjusted life year
DALY: disability adjusted life year
LFA: lateral flow antigen
GCS: Glasgow Coma Score
ULN: upper limit of normal
CRF: case-report form
SAE: severe adverse event
USAE: unexpected severe adverse event
WHO: World Health Organization
OUCRU: Oxford University Clinical Research Unit.
